# Dr. Buzby’s ToeGrips^®^ Application Results in Minimal Changes in Kinetic Gait Parameters in Normal Dogs

**DOI:** 10.3389/fvets.2017.00111

**Published:** 2017-07-13

**Authors:** James K. Roush, Leslie E. Moore, Walter C. Renberg

**Affiliations:** ^1^Department of Clinical Sciences, College of Veterinary Medicine, Kansas State University, Manhattan, KS, United States

**Keywords:** Toegrips, gait analysis, dogs, pressure plate apparatus, stance time

## Abstract

Poor traction on slick surfaces is difficult for dogs with neurologic deficits, osteoarthritis, or recovering from injury or surgery. Many dogs respond inappropriately to slick surfaces by decreasing digital pad-floor contact and extending their toenails. A device marketed to increase paw-floor friction in dogs was evaluated. Fifteen normal dogs underwent kinetic gait analysis before and after application of Dr. Buzby’s ToeGrips^®^. Ground reaction forces, including vertical peak force (VPF) and impulse for each limb, were measured and compared between pre- and post-application values. Stance time was significantly increased in all limbs after toe grip application. Stride velocity was slower in all limbs but significantly slower only in the left forelimb. VPF was significantly deceased in both hindlimbs after toe grip application, but the decrease was within the group SDs. Vertical impulse was significantly increased in both forelimbs and in the right hindlimb. Dr. Buzby’s ToeGrips^®^ result in a slower gait, with slightly decreased VPF in the hindlimbs and increased effort for propulsion kinetic changes were of minor magnitude and unlikely to be clinically relevant.

## Introduction

Poor traction on slick surfaces is a common problem in dogs, particularly for dogs that are older, arthritic, or suffering from other orthopedic or neurologic disease. Even normal dogs without orthopedic or neurologic disease experience routine caudal paw displacement, or slippage, on both non-vegetated and vegetated surfaces (1). Braking and propulsion forces, however, do not seem to be altered during compensation for the coefficient of friction of different services (2). In the authors’ clinical experience, ataxic or weak neurologic and orthopedic patients sustain new injuries, or exacerbate existing orthopedic injuries at surprising frequency secondary to slipping and falling. The frequent response of a dog to slick surfaces is to flex the digits and engage the toenails rather than the digital pads to contact the surface, a counterproductive action when the surface hardness prevents penetration by the toenails, and the smooth toenail surface result in poorer grip than the dog’s textured pads.

**Figure 1:**
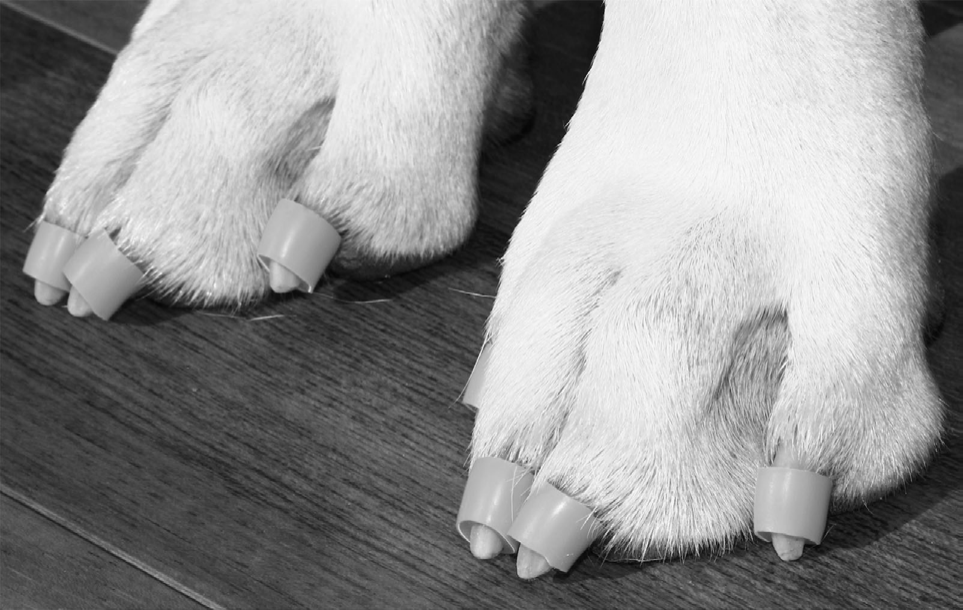
Dr. Buzby’s ToeGrips^®^ applied to a dog.

Dr. Buzby’s ToeGrips^®^ are natural rubber cylinders intended for application to a dog’s weight-bearing toenails. Toe grips are available in six sizes, XS through XXL for application based on toenail circumference to appropriately sized dogs. The toe grips cover the slick nail surface in an attempt to increase friction on smooth indoor surfaces such as tile, cement, and hardwood, and thus, provide better traction for ambulation in most dogs (Figure 1). This method of traction improvement has multiple potential veterinary medical applications, including the reduction of slipping-associated injuries in post-operative orthopedic surgery patients. If toe grips are to be employed in this manner, it is important to determine whether they detrimentally alter gait pattern, as alterations in normal post-operative gait may interfere with the normal progression of healing and clinical results. There are no previous published reports regarding the effect of Dr. Buzby’s ToeGrips^®^ on the canine gait. We hypothesized that application of Dr. Buzby’s ToeGrips^®^ would not significantly change vertical ground reaction forces and other measured gait parameters in dogs.

## Materials and Methods

Fifteen canine clinic patients with no history of lameness or inherent gait abnormalities and no evidence of orthopedic or neurologic disease on physical examination or initial pressure platform analysis were entered into the study. Study dogs were volunteered by their owners. Dogs were block assigned so that there were five small, five medium, and five large dogs examined to allow comparisons between the most common toe grip sizes. Potential exclusion criteria included dogs with nail circumferences less than 13 mm or greater than 23 mm, dogs that did not tolerate the application of toe grips, dogs that were unable to efficiently ambulate after a period of acclimation, and dogs that were unable to undergo pressure platform analysis based on temperament. The study was approved by the Kansas State University Institutional Animal Care and Use Committee.

Initial examination for inclusion into the study included a thorough history, complete physical exam, orthopedic and neurologic evaluation, and an initial force pressure platform (Hi-Rez Versatek Walkway, Tekscan Inc, South Boston, MA, USA) evaluation analyzed on commercial software (Tekscan Software, Tekscan Inc, South Boston, MA, USA). Pressure platform data collected for each dog included determination of stance time, swing time, stride time, stride length, stride velocity, vertical peak force (VPF) and impulse (normalized by percent body weight), maximum peak pressure for each limb, and a subjective description of gait and paw loading patterns that included observer assessment of changes in toe flexion and extension, dog reluctance or lack of reluctance to ambulate, and dog comfort during ambulation. Patients were walked at a comfortable velocity for each individual patient across a pressure platform centralized in a 9 m walkway covered with a 2 mm rubber mat until five complete foot strikes for each foot were obtained. Patients included in the study had their toenail circumference measured and appropriately sized Dr. Buzby’s ToeGrips^®^ (Dr. Buzby’s ToeGrips^®^ PO Box 6191, Beaufort, SC, USA) were applied to the nails of their weight-bearing digits on all four limbs. Toe grips were dipped in water-based lubricant and manually manipulated completely onto the curvature of each weight-bearing nail but without contacting the corresponding digital pad. Dogs were given 5 min of leash walk to acclimate to the toe grips as recommended by the manufacturer. The pressure platform evaluation was then repeated until five valid trials for each foot were obtained at comfortable patient-determined velocity. After evaluation, the toe grips were removed by manual traction to the dorsal surface of each toe grip.

### Statistical Analysis

Pressure platform data were averaged across all five trials before and after the toe grips and mean values for stance time, swing time, stride time, stride length, stride velocity, VPF and impulse normalized by percent body weight, and maximum peak pressure were compared by paired *t*-test between pre- and post-application values for all limbs. VPF and vertical impulse were compared between toe grip size groups in each limb by analysis of variance with a Tukey–Kramer *post hoc*. *p* ≤ 0.05 was considered significant for all comparisons.

## Results

Sixteen dogs were evaluated for the study and one dog excluded due to toenail circumference of 25 mm. Fifteen dogs completed the study. The mean weight of all dogs was 19.6 kg (median 18.2 kg, range 3.5–37.0 kg). The mean weight of dogs that received small toe grips was 9.0 kg. The mean weight of dogs that received medium toe grips was 17.6 kg. The mean weight of dogs that received large toe grips was 32.2 kg. Dog weight was significantly different between each toe grip size group (*p* < 0.001). Throughout the evaluation process, the dogs appeared comfortable and tolerated the toe grips well with rapid acclimatization. No dogs were excluded from the study after toe grip application for failure to adapt to the toe grips and ambulate in a coordinated fashion.

### All Dogs Combined

Stance time was significantly increased in all four limbs after application of the toe grips (Table 1). Swing time was significantly increased in the left forelimb only and was decreased in two of the remaining three limbs. Stride time was significantly increased in the left front and right hindlimbs and increased, but not to statistical significance, in the right forelimb. There was no significant change in stride length before or after toe grip application. Stride velocity was slower in all limbs, but significantly slower only in the left forelimb after toe grip application. VPF normalized for body weight was significantly deceased in both hindlimbs after application of toe grips VPF in the forelimbs was not significantly changed by application of toe grips. Vertical impulse was significantly increased in both forelimbs and in the right hindlimb after toe grip application. Vertical impulse was also increased in the left hindlimb but not significantly. There were no significant changes in maximum peak pressure after application of the toe grips. No subjective differences in gait or paw-loading pressure patterns were noted.

**Table 1:** Mean data (±SD) pre- and post-application of Dr. Buzby’s ToeGrips^®^.

Parameter	Limb	Mean pre	Mean post	% change
Weight (kg)		19.59 ± 11.26		
Stance time (s)	LF	0.29 ± 0.46	0.33 ± 0.12*	13.7
	LH	0.26 ± 0.42	0.30 ± 0.13*	15.5
	RF	0.30 ± 0.44	0.33 ± 0.12*	11.2
	RH	0.27 ± 0.44	0.32 ± 0.14*	21.1
Swing time (s)	LF	0.22 ± 0.05	0.24 ± 0.04*	7.2
	LH	0.33 ± 0.22	0.29 ± 0.08	
	RF	0.24 ± 0.05	0.23 ± 0.07	
	RH	0.29 ± 0.15	0.30 ± 0.13	
Stride time (s)	LF	0.52 ± 0.14	0.57 ± 0.15*	8.3
	LH	0.60 ± 0.27	0.60 ± 0.19	
	RF	0.53 ± 0.13	0.57 ± 0.16	
	RH	0.56 ± 0.19	0.61 ± 0.22*	8.6
Stride length (cm)	LF	73.83 ± 17.06	70.43 ± 15.84	
	LH	70.79 ± 17.79	71.44 ± 14.33	
	RF	73.81 ± 17.35	72.41 ± 17.01	
	RH	71.82 ± 17.27	71.81 ± 15.68	
Stride velocity (cm/s)	LF	145.97 ± 29.20	129.17 ± 31.10*	−11.5
	LH	130.51 ± 39.87	128.44 ± 35.69	
	RF	143.21 ± 28.62	132.53 ± 29.97	
	RH	137.85 ± 36.58	126.56 ± 34.06	
Vertical peak force kg/%BW	LF	57.15 ± 10.64	56.89 ± 10.23	
	LH	39.99 ± 6.70	37.09 ± 6.31*	−7.3
	RF	59.01 ± 12.89	59.41 ± 11.47	
	RH	38.32 ± 7.21	36.46 ± 6.48*	−4.9
Impulse kg/%BW	LF	10.27 ± 3.54	11.71 ± 4.22*	14.2
	LH	6.59 ± 2.41	6.91 ± 2.85	
	RF	10.28 ± 3.46	11.65 ± 4.15*	13.3
	RH	6.36 ± 2.69	6.85 ± 2.92*	7.7
Max peak pressure (kg/cm^2^)	LF	1.13 ± 0.39	1.11 ± 0.40	
	LH	0.96 ± 0.33	0.95 ± 0.33	
	RF	1.09 ± 0.36	1.11 ± 0.40	
	RH	0.97 ± 0.40	0.94 ± 0.32	

*Indicates significant difference at p < 0.05 after toe grip application. % change is only noted for significantly different values.

### Toe Grip Size

Vertical peak force normalized for body weight was not significantly different for any limb between small, medium, and large toe grip-sized dogs either before or after toe grip application (Table 2). Vertical impulse normalized for body weight increased from small to large toe grip dogs with multiple points of significant difference as noted in Table 2.

**Table 2:** Comparison of normalized forces on each limb between small, medium, and large dogs before and after application of Dr. Buzby’s ToeGrips^®^.

Parameter	Leg	Small pre	Small post	Medium pre	Medium post	Large pre	Large post
VPF (kg/%BW)	LF	60.0 ± 14.0	58.68 ± 14.9	56.1 ± 9.6	58.04 ± 9.9	55.4 ± 9.5	53.96 ± 5.6
	RF	64.88 ± 17.2	62.56 ± 16.9	56.92 ± 9.4	60.54 ± 9.5	55.22 ± 11.5	55.12 ± 7.0
	LH	43.16 ± 9.9	37.02 ± 9.0	38.56 ± 4.2	38.90 ± 6.8	38.26 ± 4.7	35.34 ± 2.3
	RH	37.16 ± 10.7	34.86 ± 8.6	39.42 ± 6.5	39.72 ± 7.2	38.38 ± 4.7	34.8 ± 2.3
VI (kg/%BW)	LF	7.10 ± 1.0^a^	8.20 ± 2.7^a^	10.26 ± 3.3^ab^	10.70 ± 3.3^a^	13.44 ± 2.6^b^	16.22 ± 1.5^b^
	RF	7.22 ± 0.8^b^	8.02 ± 2.4^a^	10.32 ± 3.2^ab^	10.84 ± 3.2^a^	13.30 ± 2.8^b^	16.08 ± 1.5^b^
	LH	4.44 ± 1.0^a^	4.44 ± 1.4^a^	6.30 ± 2.2^a^	6.28 ± 2.3^a^	9.04 ± 1.0^b^	10.02 ± 1.0^b^
	RH	3.44 ± 0.6^a^	4.09 ± 1.6^a^	6.40 ± 1.8^b^	6.60 ± 2.1^b^	9.24 ± 0.8^c^	9.94 ± 0.8^c^

*There are no significant differences in vertical peak force (VPF) between small, medium, and large dogs. Vertical impulse (VI) values that are not followed by the same superscript letters are significantly different between toe grip size groups within the pre or post time period*.

## Discussion

After application of Dr. Buzby’s ToeGrips^®^ and a short acclimation period, stance time was significantly increased in all dogs, and there was a corresponding decrease in stride velocity, but no significant change in stride length was seen. Coupled with subjective impressions of the gait and pressure patterns, we conclude that toe grips result in a slower normal gait preference by the animal, but no change in pattern. It is not surprising that a gait pattern change was not observed, since essentially the toe grips change friction and surface contact characteristics for the dog and previous kinetic gait analysis of healthy dogs showed no differences on ground reaction forces between linoleum and carpet (2).

The slight but statistically significant decrease in hindlimb VPF along with a corresponding increase in stance time and decreased stride velocity suggests that dogs are striking the hindlimb slower. The decreased hindlimb VPF is thus likely artificial due to the slower stance time and slower velocity as demonstrated in other studies (3, 4). Additionally, the hindlimb decreases of 2.90%BW in the left hindlimb and 1.86% body weight in the right hindlimb after toe grip application are well within the standard deviations of the pre- and post-application values and thus are unlikely to reflect clinically significant changes in ambulation. Significant increases in the vertical impulse of the left and right forelimbs and right hindlimb suggest that either the dogs may be pushing off more in an attempt to maintain velocity or that the softer rubber toe grips result in an need for increase in vertical impulse for propulsion, or both.

Vertical peak force normalized to body weight did not change with increasing size, while vertical impulse increased with increasing body weight and toe grip size despite normalizing the force for body weight. These findings have been noted before in other studies assessing body size and dog breed (5, 6). We did not analyze kinetic parameters that were not normalized for body weight, such as stance time and stride length, because these would obviously be different between dogs of different sizes.

The period of acclimation in these dogs was short and as suggested by the manufacturer. Dogs adapted quickly and were able to ambulate with visibly normal gaits soon after application. It is possible longer periods of acclimation might result in even less changes in ground reaction forces, or alternatively that a greater accommodation in gait might be necessary.

Ground reaction forces at the shoe-floor interface are likely the most critical biomechanical factor in slips in people (7, 8). Floor slipperiness is one of the critical factors affecting the risk of slipping and falling. The optimum coefficient of friction to prevent slips in dogs is unknown, and we did not measure the change in coefficient of friction created by the application of toe grips in these dogs. Precise determination of coefficient of friction varies widely by the measurement tool and the static versus dynamic motion of the subject (9). Pigs walking on slippery floors lower their walking speed and prolong their stance phase duration on greasy compared with dry and wet concrete (10). That response is similar to the changes caused by the toe grips in this study and the adaptations in both cases presumably increase the confidence of the animal in their gait stability. Dogs on slick surfaces flex their digits and engage toenails rather than digital pads to contact the surface, thus presumably decreasing the dynamic coefficient of friction similar to the manner in which hard-soled shoes provide less available friction than soft-soled shoes in people (11). The gait and force changes seen in this study after toe grip application counter the natural tendencies of dogs on slip surfaces and should result in better stability. Because we did not evaluate changes in the coefficient of friction created by the toe grips, further gait and clinical studies are needed to objectively and directly assess those changes. Such studies will be difficult to carry out since slick surfaces are often unyielding and unyielding surfaces prevent concurrent measurement of ground reaction forces. Further studies of toe grip application in dogs that have lameness from naturally occurring osteoarthritis or mild neurologic disease are also needed because dogs in this study were normal dogs without preexisting disease.

The authors believe that the changes observed in this study are unlikely to be clinically significant to the mobility or comfort of normal dogs. Application of Dr. Buzby’s ToeGrips^®^ results in minimal changes to kinetic gait parameters. Further studies are needed to directly measure changes in the coefficient of friction after toe grip application and to determine if dogs with impaired gait (such as postsurgical orthopedic patients, dogs with osteoarthritis, or dogs with mild neurologic deficits) will benefit from toe grip application.

## Ethics Statement

The study was approved by the Kansas State University Institutional Animal Care and Use Committee.

## Author Contributions

All listed authors were involved in data collection, manuscript preparation, manuscript approval and are accountable for all aspects of the work.

## Conflict of Interest Statement

The authors declare that the research was conducted in the absence of any commercial or financial relationships that could be construed as a potential conflict of interest.
